# Trabecular bone score as a possible new surgical recommendation in patients with asymptomatic primary hyperparathyroidism

**DOI:** 10.1007/s40618-025-02590-9

**Published:** 2025-04-25

**Authors:** Nicolò Bisceglia, Matteo Malagrinò, Cristiana Cantone, Sara Capra, Anna Piazza, Giulia Vandi, Guido Zavatta

**Affiliations:** 1 Division of Endocrinology and Diabetes Prevention and Care, IRCCS Azienda Ospedaliero- Universitaria, Bologna, Italy; 2 Department of Medical and Surgical Sciences (DIMEC), Alma Mater Studiorum University of Bologna, Via Massarenti, 9, Bologna, 40138 Italy

**Keywords:** Primary hyperparathyroidism, TBS, Parathyroidectomy, Fracture risk, Guidelines, Fifth International Workshop

## Abstract

**Purpose:**

TBS usefulness in primary hyperparathyroidism (PHPT) management is uncertain. The primary aim was to evaluate the significance of performing Trabecular Bone Score (TBS) in addition to classical PHPT surgical recommendations. The explorative objective was to characterize bone quality in a cohort of patients with asymptomatic PHPT.

**Methods:**

From 1/1/2021 to 5/31/2024, of 265 consecutively evaluated PHPT patients, 125 met inclusion and exclusion criteria. Patients underwent complete biochemical evaluation in the same laboratory, Dual Energy X-ray Absorptiometry (DXA) at three sites, TBS with the same densitometer, and renal ultrasound. We retrospectively applied to each patient all the criteria to recommend parathyroidectomy as per the Fifth International Workshop, adding also TBS ≤ 1.2.

**Results:**

Almost one-third of patients presented degraded TBS (≤ 1.2), 36.0% partially degraded TBS, and 31.2% preserved TBS (≥ 1.35). Mean TBS was partially degraded (1.286 ± 0.134). Osteoporotic patients had lower TBS than osteopenic patients (1.207 ± 0.101 vs. 1.27 9 ± 0.110; *p* <.001). Patients with degraded TBS had a greater number of fragility fractures than those with TBS > 1.2 (34.2% vs. 8.3%; *p* <.001). In the group of osteopenic patients (34.4%), 10/43 (23.3%) showed degraded TBS. 13 patients (10.4%) did not meet any surgical criteria, and of these, 3/13 (23.1%) had degraded TBS and were osteopenic.

**Conclusion:**

More than one-fifth of patients who did not meet the current surgical recommendations presented degraded TBS and osteopenia. The combination of degraded TBS and osteopenia as a possible new surgical recommendation could increase the number of patients who might benefit from curative parathyroidectomy, with potential reduction of fracture risk.

## Introduction

Primary hyperparathyroidism (PHPT) is the most common cause of hypercalcemia in outpatients, and it is defined as an increased and inappropriate oversecretion of parathyroid hormone by one or more parathyroid glands in relation to the levels of serum calcium (sCa) [1]. In recent decades, there has been a gradual change in PHPT clinical presentation from frankly symptomatic to more asymptomatic phenotypes, especially owing to the introduction of routine metabolic screening including sCa [2]. For these reasons, PHPT is now more frequently diagnosed incidentally in asymptomatic post-menopausal women or in symptomatic patients due to fragility fractures [3, 4]. It is known that the catabolic effects of PTH excess are initially shown in the cortical bone, however, a densitometric pattern with preferential involvement of the lumbar spine has also been reported, which may only partially be explained by the early effects of physiological estrogen deficiency in postmenopausal women before the onset of PHPT [5].

Recent epidemiological data have suggested an increased risk of both vertebral and peripheral fractures in PHPT [6]. The paradox of increased risk of vertebral fractures in PHPT despite relatively preserved lumbar spine Bone Mineral Density (BMD) has remained unclear until recently, with the introduction of new non-invasive imaging techniques to assess bone microarchitecture such as HRpQCT (High-resolution peripheral quantitative computed tomography) and TBS (Trabecular bone score).

Specifically, HRpQCT is a non-invasive technique that assesses trabecular and cortical microstructure of the distal radius and tibia, and recent studies [7, 8] have shown that microarchitecture in both cortical and trabecular bone is degraded in PHPT patients, which may explain the increased incidence of vertebral fragility fractures in patients with this disorder. Unfortunately, HRpQCT is expensive and not widely available in the clinical setting.

TBS is much more widespread in clinical practice due to its low invasiveness, low cost and ease of image acquisition. TBS consists of a variation of the gray scale in a two-dimensional projection obtained by DXA, and it is calculated using TBS iNsight^®^, a software that, through the analysis of lumbar densitometry, allows to estimate fracture risk based on bone microarchitecture [9]. TBS is not a direct measure of bone microarchitecture but it is correlated with three-dimensional features such as the number of trabeculae, trabecular separation and the density of connectivity [10, 11]. TBS has been the subject of many studies in recent years, according to which it has been suggested that a low TBS value is associated with an increased incidence of vertebral fractures both in healthy patients [12] and in those with endocrine diseases [13], such as PHPT [14]. Furthermore, TBS has been shown to be largely independent from BMD and from FRAX, and when used in conjunction with either one of these measures, it consistently enhances their accuracy [15].

The evaluation of bone microarchitecture therefore seems relevant to refine the estimate of fracture risk in PHPT, and to specifically detect potential candidates for parathyroid surgery, who cannot otherwise be selected by a standard bone density test alone. For the above-mentioned reasons, we decided to conduct this study, in order to highlight the proportion of patients with relatively preserved BMD, yet with degraded bone quality, who could be worthy of surgery only for this last feature. The inclusion of TBS among the surgical criteria could expand the number of high-risk patients to benefit from parathyroidectomy. Moreover, this article comes timely to address the research agenda promoted by the Fifth International Workshop [16], advocating the need for further data on the clinical utility of TBS in the management of PHPT.

## Objectives

Primary aim: to clarify the usefulness of TBS in clinical practice, proposing it as a means for identifying a proportion of PHPT patients at high fracture risk who could benefit from parathyroidectomy. Therefore, to evaluate whether degraded TBS could be considered as a possible new indication for surgical treatment of primary hyperparathyroidism beyond the standard criteria.

Explorative aim: to better characterize bone quality in PHPT patients, through the description of the prevalence of low TBS and also comparing clinical, biochemical and densitometric features in subpopulations with different bone quality.

## Patients and methods

### Ethics

This study was conducted in line with STROBE (STrengthening the Reporting of OBservational studies in Epidemiology) recommendations, and all patients consented to participate. The Ethical Committee of our Hospital (CE-AVEC) approved this study (protocol code: PARAT-970/2021/Oss/AOUBo).

### Study design and population

From January 1 st, 2021, through May 31 st, 2024, 265 patients with an established diagnosis of PHPT were consecutively seen at the Parathyroid and Bone Metabolism outpatient clinic of the Endocrinology and Prevention and Care of Diabetes Unit of IRCCS Azienda Ospedaliero-Universitaria of Bologna, Italy. After the application of inclusion and exclusion criteria (s. below), we selected 125 patients and we retrospectively applied to each patient all the criteria to recommend parathyroid surgery as per the Fifth International Workshop [16].

This is a single-center, observational, retrospective study.

### Inclusion criteria

Patients with asymptomatic hypercalcemic PHPT (PHPT definition: as per V International Workshop [16]) who had a DXA at three sites performed with the same machine (Ge-Lunar iDXA^®^) within 6 months before the visit. TBS value was calculated in each DXA, and bone microarchitecture was considered degraded when this was ≤ 1.2, while it was considered partially degraded when it was between 1.2 and 1.35 and preserved if TBS was ≥ 1.35.

All patients underwent the same laboratory tests in the LUM - Unified Metropolitan Laboratory of Bologna (www.ausl.bologna.it/servospe/lum*)* - within 1 month before the scheduled visit, as part of pre-specified clinical outpatient protocols: PTH (reference range: 12–88 pg/mL), serum total calcium (sCa) (reference range: 8.6–10.5 mg/dL), serum phosphate (reference range: 2.5–4.5 mg/dL), 25(OH)D (reference range: 20–100 ng/mL), albumin (reference range: 35–50 g/L), serum creatinine (reference range: 0.5–1.2 mg/dL), 24-hour urinary calcium (reference range: 50–400 mg/die), 24-hour urinary phosphorus (reference range: 0.4–1.3 g/die), serum bone alkaline phosphatase (reference range: 5.5–27.1 microg/L), serum C-terminal telopeptide of collagen type 1 (CTX) (reference range: 0.142–1.351 ng/mL), ionized calcium (reference range: 1.15–1.29 mmol/L). Hypercalciuria was defined as > 250 mg/day in women or > 300 mg/day in men [16]. Abdominal ultrasound was also a part of endocrinological work-up to assess the presence of nephrolithiasis or nephrocalcinosis. Morphometric X-ray of the dorsal-lumbar spine, chest X-ray or scout CT scan were reviewed to investigate the presence of morphometric vertebral fractures.

### Exclusion criteria

We excluded patients with any known condition that can affect bone and calcium metabolism, such as familial hypocalciuric hypercalcemia, secondary hyperparathyroidism, previous parathyroidectomy, Paget’s disease of the bone or other diseases known to affect bone metabolism (thyrotoxicosis, bowel diseases, chronic hepatic disease, hypercortisolism, rheumatological or hematological diseases) and patients taking drugs potentially interfering with blood and urine calcium levels (cinacalcet, denosumab, zoledronate, thiazide diuretics, furosemide, proton-pump inhibitors). Patients on oral bisphosphonates were retained when administered for up to two years, because no effect on sCa, nor a significant effect on TBS have been demonstrated with these medications [4].

Patients with comorbidities that could potentially alter bone quality, such as diabetes mellitus, breast cancer, chronic autoimmune gastritis, inflammatory bowel disease (IBD) and lymphoma, were also excluded.

### Statistics

Absolute numbers and percentages were calculated for categorical data. The results for continuous variables were expressed as means and standard deviation (SD), minimum and maximum. A comparison of cases and controls was performed by Mann-Whitney U test. χ2 test was used to detect associations between categorical data. The sample had the statistical power to estimate proportions with an error margin of 8.75%, based on a sample of 125 individuals. Univariate analysis between subgroups of TBS were performed using age and BMI as covariates. Statistical analyses were performed using SPSS (version 30.0). P Values less than 0.05 were considered statistically significant.

## Results

### Clinical characteristics and bone mineral status

**Fig. 1:**
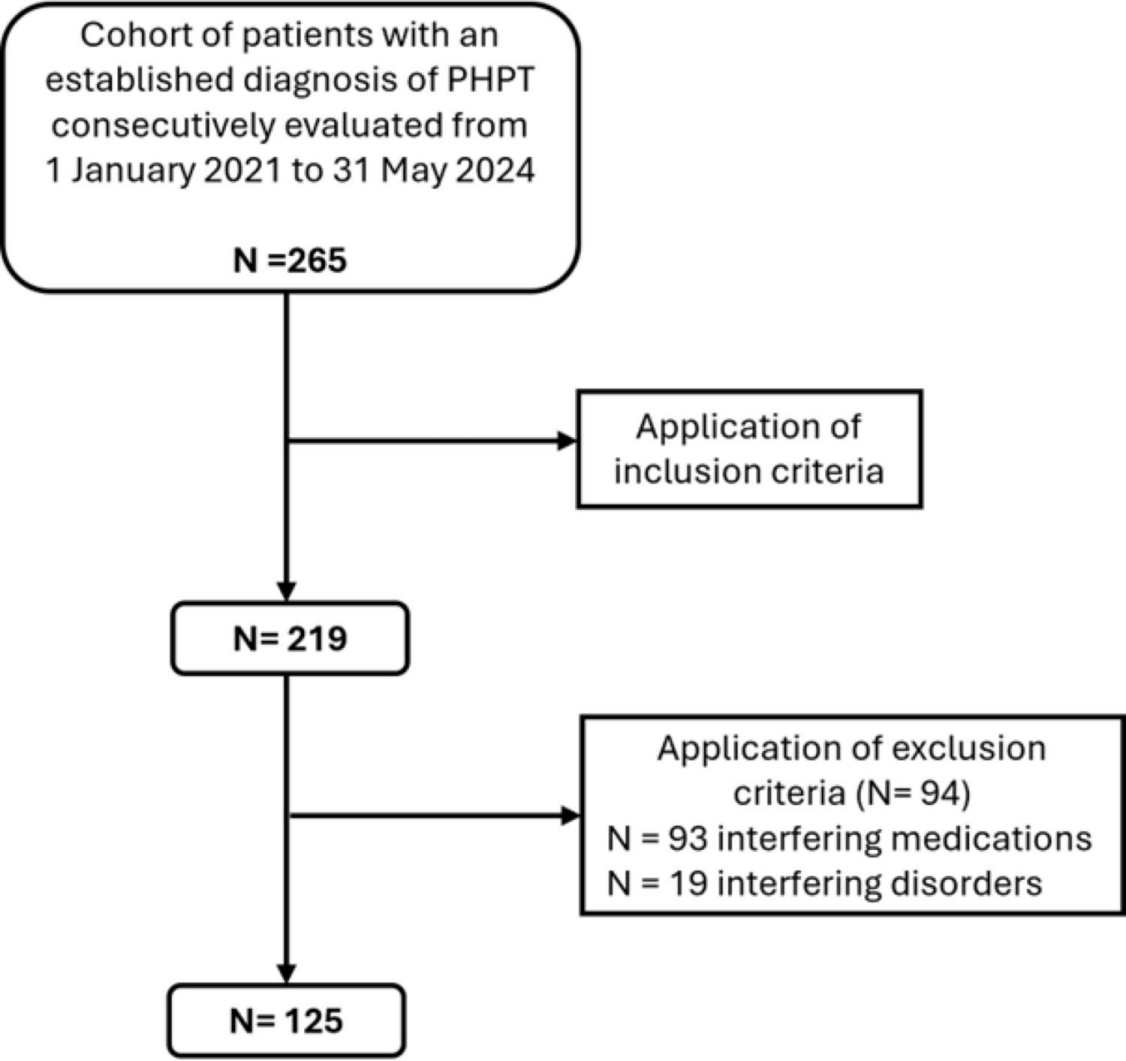
Patient selection process

**Fig. 2:**
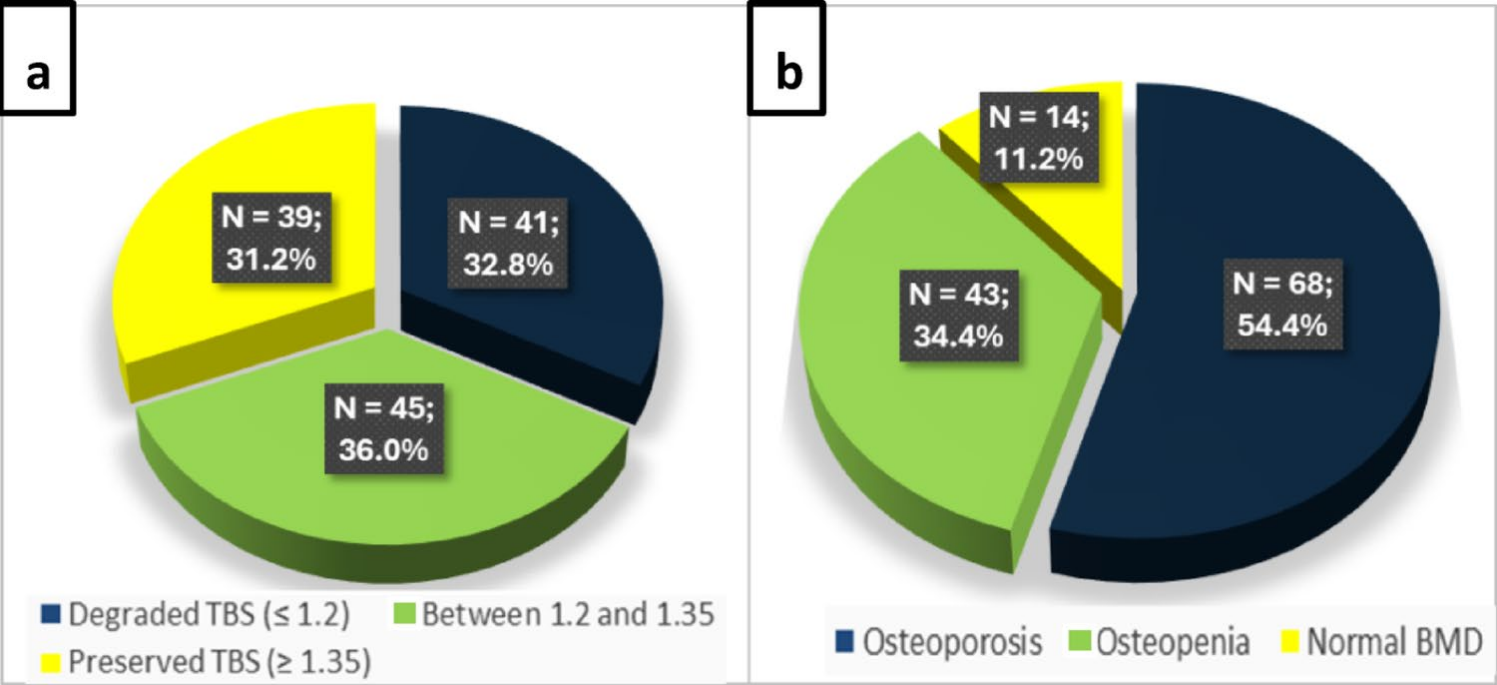
TBS (**a**) and BMD (**b**) in study population (*N* = 125). TBS, Trabecular bone score; BMD, Bone Mineral Density.

After the application of inclusion and exclusion criteria (see above), the study population included 125 patients with PHPT (Fig. 1). Our population was predominantly composed of women (84.8%), mean age was 64.4 years, mean BMI was 26.9 kg/m^2^ and 54 patients (43.2%) suffered from blood hypertension (Table 1). As regards radiological features (Fig. 2), osteoporotic patients were the majority (*N* = 68; 54.4%), patients with osteopenia were 43 (34.4%), and those with normal BMD were 14 (11.2%). Patients with degraded TBS were 41 (32.8%), those with partially degraded TBS were 45 (36%) and those with preserved TBS (≥ 1.35) were 39 (31.2%). We classified PHPT patients into two subgroups (Table 1): 41(32.8%) with degraded TBS and 84 (67.2%) with normal or partially degraded TBS. Patients with degraded TBS were significantly older than their counterparts with TBS > 1.2 (69.0 years vs. 62.1 years) and were more commonly women (95.1% vs. 79.8%). The lumbar spine T-score of the whole population was − 1.6 and mean TBS value was partially degraded at 1.286 ± 0.134. Patients with degraded TBS had a lower lumbar spine T-score than patients with TBS > 1.2 (−2.5 vs. −1.1; *p* <.001). Mean femoral neck and total hip T-scores of the whole study population were in the osteopenic range (−1.8 and − 1.5, respectively), similar to what occurred in ultradistal radius and one-third distal radius BMD T-scores (−1.8 and − 1.9, respectively). Patients with degraded TBS had also significantly lower T-scores than patients with TBS > 1.2 at these sites.

**Table 1:** Clinical characteristics and bone mineral status of the study population according to TBS groups

Variables	Study population *N* = 125	TBS > 1.2 *N* = 84	TBS ≤ 1.2 *N* = 41
Age [years]	64.4 ± 11.4 [26; 87]	62.1 ± 12.0^*^ [26; 87]	69.0 ± 8.5 [47; 85]
Sex [F(%)/M(%)]	106 (84.8%)/19 (15.2%)	67(79.8%)/17(20.2%)^*^	39(95.1%)/2(4.9%)
BMI [kg/m^2^]	26.9 ± 5.2 [17.7; 42.6]	27.4 ± 5.2 [17.7; 38.4]	26.0 ± 4.9 [18.2; 42.6]
Arterial Hypertension, n (%)	54 (43.2%)	36 (42.9%)	18 (43.9%)
Lumbar Spine BMD [g/cm^2^]	0.995 ± 0.215 [0.246; 1.720]	1.057 ± 0.205^**^ [0.705; 1.720]	0.868 ± 0.177 [0.246: 1.174]
Lumbar Spine T-score	−1.6 ± 1.6 [−5.1; 3.7]	−1.143 ± 1.549^**^ [−3.9; 3.7]	−2.5 ± 1.2 [−5.1; −0.1]
Lumbar Spine Z-score	−0.5 ± 1.4 [−3.3; 3.6]	−0.3 ± 1.4^*^ [−2.9; 3.6]	−1.0 ± 1.1 [−3.3; 1.7]
TBS (L1-L4)	1.286 ± 0.134 [1.020; 1.584]	1.355 ± 0.106^**^ [1.201; 1.584]	1.146 ± 0.046 [1.020; 1.198]
TBS (L1-L4) T-score	−1.9 ± 1.4 [−4.7; 1.1]	−1.2 ± 1.1^**^ [−2.9; 1.1]	−3.4 ± 0.5 [−4.7; −2.2]
Femoral Neck BMD [g/cm^2^]	0.774 ± 0.124 [0.525; 1.092]	0.805 ± 0.129^**^ [0.553; 1.092]	0.711 ± 0.082 [0.525; 0.836]
Femoral Neck T-score	−1.8 ± 0.9 [−3.8; 0.4]	−1.6 ± 0.9^**^ [−3.6; 0.4]	−2.3 ± 0.7 [−3.8; −1.3]
Femoral Neck Z-score	−0.6 ± 0,7 [−2.7; 1.0]	−0.6 ± 0.7 [−2.3; 1.0]	−0.7 ± 0.7 [−2.7; 0.6]
Total Hip BMD [g/cm^2^]	0.825 ± 0.146 [0.472; 1.241]	0.862 ± 0.155^**^ [0.472; 1.241]	0.748 ± 0.083 [0.576; 0.951]
Total Hip T-score	−1.5 ± 1.1 [−3.7; 1.2]	−1.2 ± 1.1^**^ [−3.7; 1.2]	−2.1 ± 0.7 [−3.5; −0.4]
Total Hip Z-score	−0.5 ± 0.9 [−2.7; 1.8]	−0.4 ± 0.9^*^ [−2.4; 1.8]	−0.8 ± 0.7 [−2.7; 0.7]
Ultradistal radius BMD [g/cm^2^]	0.395 ± 0.114 [0.204; 0.941]	0.426 ± 0.116^**^ [0.204; 0.941]	0.331 ± 0.082 [0.222; 0.568]
Ultradistal radius T-score	−1.8 ± 2.1 [−5.8; 3.3]	−1.2 ± 2.0^**^ [−5.8; 3.3]	−3.0 ± 1.7 [−5.4; 0.9]
Ultradistal radius Z-score	−0.5 ± 1.8 [−4.6; 4.3]	−0.2 ± 1.8^*^ [−4.6; 4.3]	−1.2 ± 1.5 [−3.6; 1.6]
One-third distal radius BMD [g/cm^2^]	0.722 ± 0.157 [0.430; 1.042]	0.762 ± 0.156^**^ [0.430; 1.042]	0.640 ± 0.127 [0.462; 0.884]
One-third distal radius T-score	−1.9 ± 1.6 [−5.1; 1.2]	−1.5 ± 1.6^**^ [−5.1; 1.2]	−2.7 ± 1.4 [−4.7; 0.1]
One-third distal radius Z-score	−0.7 ± 1.3 [−4.0; 2.8]	−0.5 ± 1.3^*^ [−4.0; 2.8]	−1.1 ± 1.3 [−4.0; 1.

BMI Body mass index; TBS, Trabecular bone score; BMD, Bone Mineral Density. Mean ± Standard Deviation [Min; Max].

**P <.001 vs. Degraded TBS; *P <.05 vs. Degraded TBS.

§§P adjusted for age and BMI < 0.001 vs. Degraded TBS; § P adjusted for age and BMI < 0.05 vs. Degraded TBS.

**Fig. 3:**
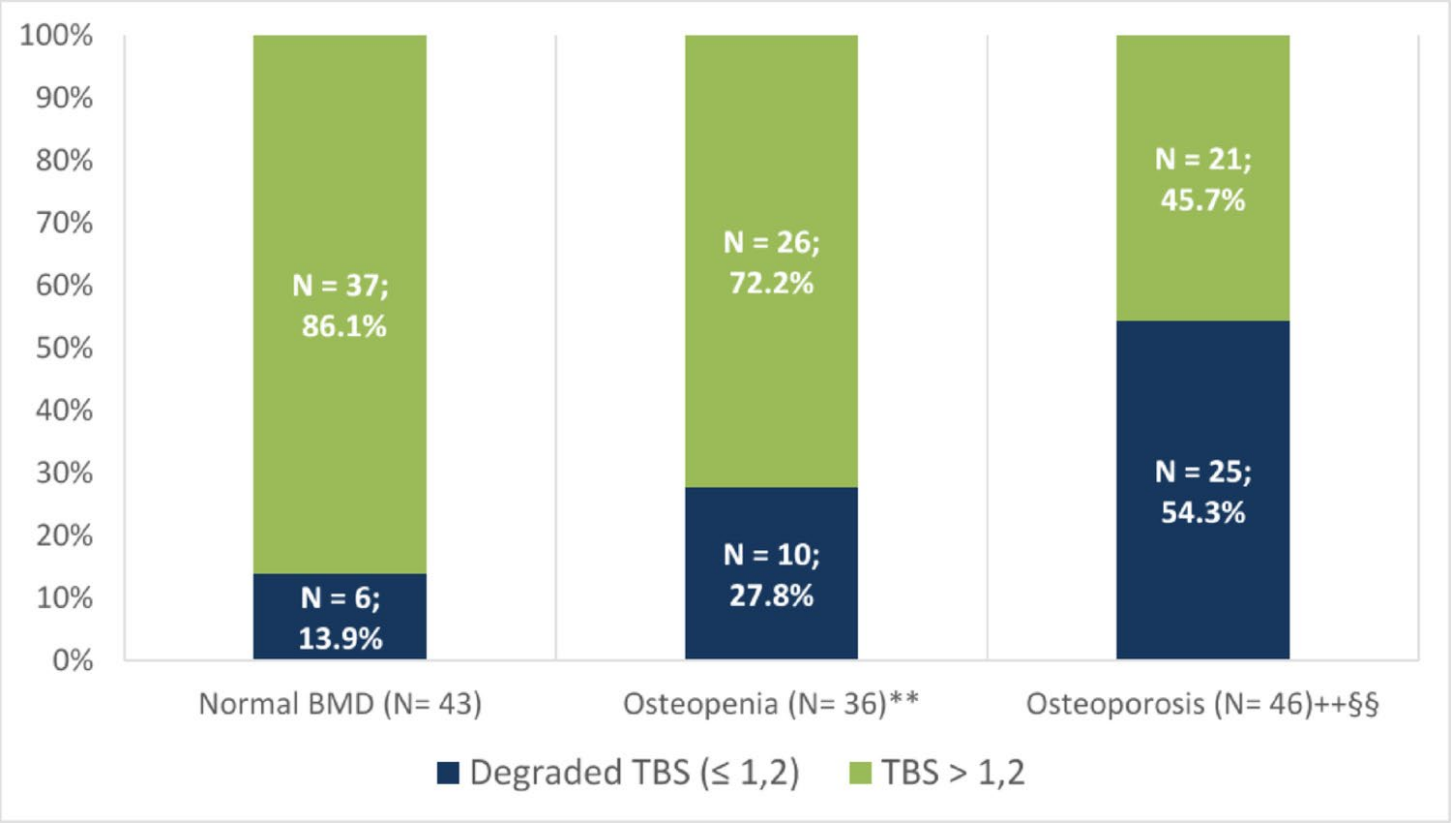
TBS distribution according to lumbar spine BMD. TBS, Trabecular bone score; BMD, Bone Mineral Density.**P<.001 vs Normal BMD; *P <.05 vs Normal BMD; ++P <.001 vs Normal BMD; +P <.05 vs Normal BMD;§§P <.001 vs Osteopenia; §P <.05 vs Osteopenia.

Classifying patients according to DXA categories of the lumbar spine (Fig. 3), we found that there were 46 patients (36.8%) with osteoporosis, 36 (28.8%) with osteopenia and 43 (34.4%) with normal BMD. The number of patients with degraded TBS was significantly higher (54.3%) in osteoporotic patients than both osteopenic (27.8%) and normal lumbar spine BMD patients (13.9%).

**Fig. 4:**
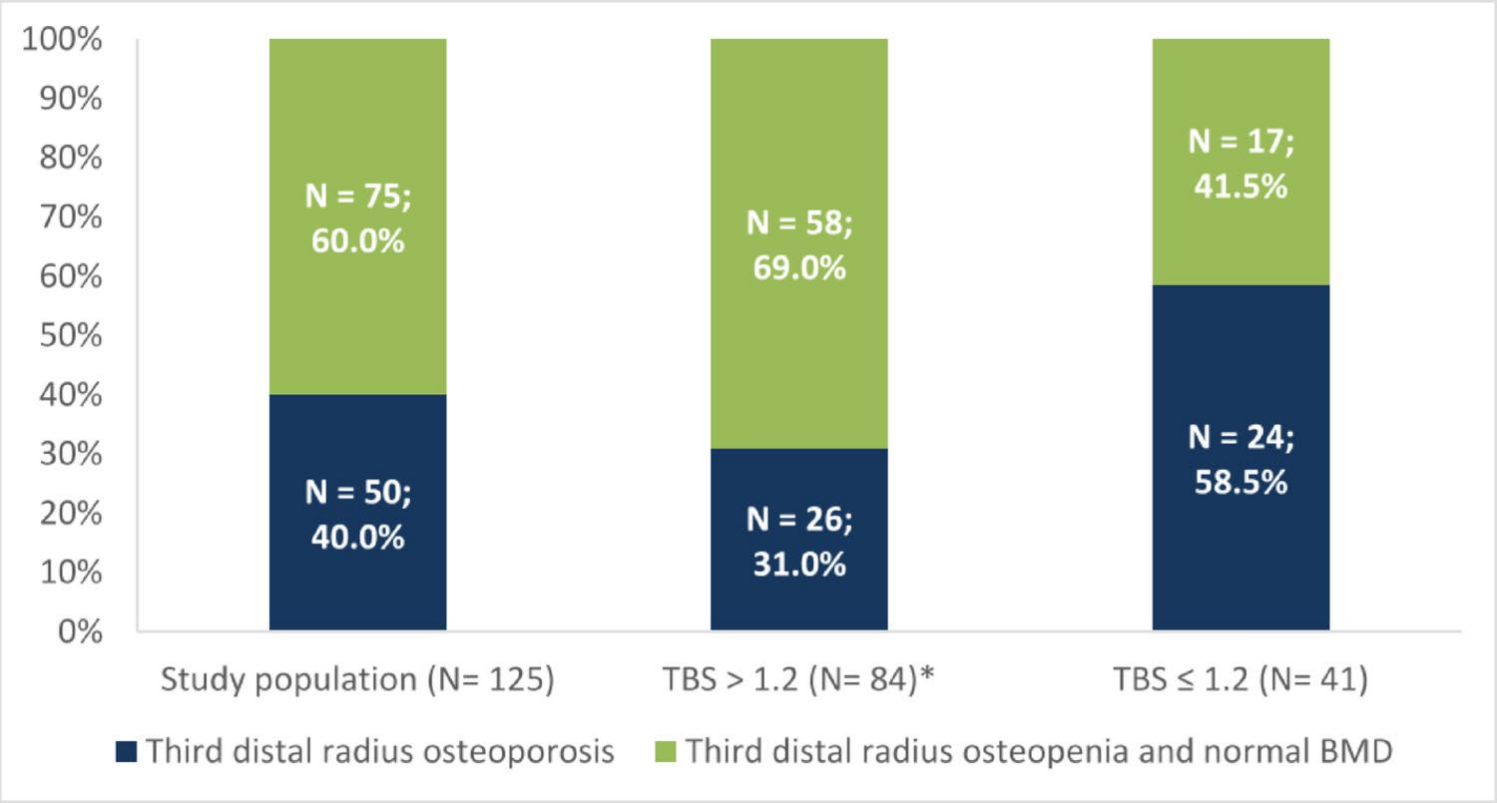
TBS in patients with third distal radius osteoporosis. TBS, Trabecular bone score.*P =.003 vs degraded TBS (≤ 1,2).

Patients with third distal radius osteoporosis were 50 (40%) in the whole study population (Fig. 4), in particular the subgroup with degraded TBS had a significantly higher incidence of osteoporosis at third distal radius than those with TBS > 1.2 (58.5% vs. 31.0%, *p* =.003).

We then analyzed patients with osteoporosis (*N* = 68) focusing on the differences in terms of clinical and morphometric fractures between those with degraded TBS (*N* = 31) and non-degraded TBS (*N* = 37).

As for clinical fractures, the comparison was very close to significance (*p* =.057), in fact, in the cohort of patients with osteoporosis and degraded TBS, 9 patients had clinical fractures (29.0%), while those with osteoporosis and non-degraded TBS had a smaller number of clinical fractures (*N* = 4; 10.8%).

Regarding morphometric fractures, these were comparable between the two subgroups (*p* =.356), in fact only one patient with osteoporosis and degraded TBS had a morphometric vertebral fracture, while no patient in the other subgroup.

The same analysis in those with normal BMD (*N* = 14) could not be performed, because none of the patients with normal BMD presented degraded TBS.

### Parathyroid surgery criteria

The most frequent complications in our PHPT cohort were osteoporosis (54.4%) and hypercalciuria (49.6%) (Table 2). The other surgical indications, in order of frequency, were nephrolithiasis (25.6%), serum calcium 1 mg/dl above the upper normal limit (24.0%), any previous clinical fragility fracture or morphometric vertebral fracture (16.8%), age less than 50 years (13.6%) and reduced GFR (< 60 ml/min) (7.2%). A group of 13 patients (10.4%) did not meet any surgical criteria.

**Table 2:** Surgical criteria in the whole population and according to TBS categories

Variables	Study population *N* = 125	TBS > 1.2 *N* = 84	TBS ≤ 1.2 *N* = 41
Age < 50 years, n (%)	17 (13.6%)	15 (17.8%)^*^	2 (4.9%)
Fragility fracture or morphometric vertebral fracture, n (%)	21 (16.8%)	7 (8.3%)^**^	14 (34.2%)
Serum calcium > 1 mg/dL above upper limit of normal, n (%)	30 (24.0%)	21 (25.0%)	9 (22.0%)
Reduced BMD by DXA to a T-score of ≤ − 2.5 at any site, n (%)	68 (54.4%)	37 (44.1%)^*^	31 (75.6%)
Creatinine clearance < 60 mL/min, n (%)	9 (7.2%)	3 (3.6%)^*^	6 (14.6%)
Kidney stones or nephrocalcinosis by abdominal imaging, n (%)	32 (25.6%)	19 (22.6%)	13 (31.7%)
Hypercalciuria (> 250 mg/day for females or > 300 mg/day for males), n (%)	62 (49.6%)	50 (59.2%)^*^	12 (29.3%)
Not meeting any surgical criteria, n (%)	13 (10.4%)	10 (11.9%)	3 (7.3%)
Osteopenia and degraded TBS	10 (8.0%)	NA	NA

TBS, Trabecular bone score; BMD, Bone Mineral Density; DXA, Dual Energy X-ray Absorptiometry.

**P <.001 vs. Degraded TBS; *P <.05 vs. Degraded TBS.

**Fig. 5:**
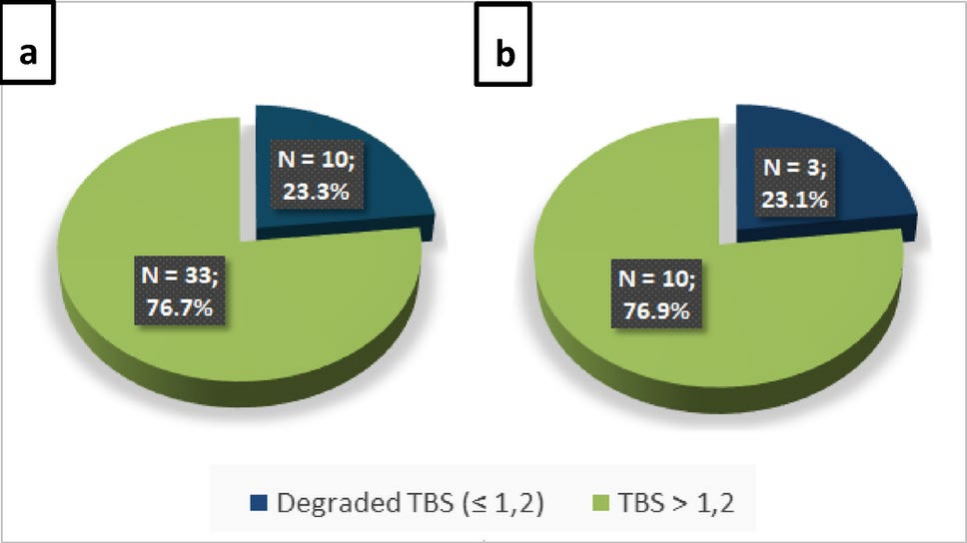
TBS in osteopenic patients (*N* = 43) (**a**) and in patients not meeting any surgical criteria (*N* = 13) (**b**). TBS, Trabecular bone score.

Considering TBS within the various surgical indications, it was noted that patients with degraded TBS had a greater number of fragility fractures, including morphometric vertebral fractures, than those with TBS > 1.2 (34.2% vs. 8.3%, *p* <.001), as well as a higher incidence of osteoporosis (75.6% vs. 44.1%, *p* <.05) and reduced renal function (14.6% vs. 3.6%, *p* <.05). Patients with TBS > 1.2 were younger (age < 50 years) than those with degraded TBS (17.8% vs. 4.9%, *p* <.05) and had a higher incidence of hypercalciuria (59.2% vs. 29.3%, *p* <.05). No differences were found between kidney stone prevalence and serum calcium levels between TBS subgroups. When we assessed the prevalence of degraded TBS in osteopenic patients and in those who did not meet any surgical criteria **(**Fig. 5**)**, it was found that 23.3% of osteopenic patients and 23.1% of patients who did not meet the current recommendations had degraded TBS. Of note, patients who did not meet any surgical criteria with degraded TBS were all osteopenic.

### Biochemical features

Regarding biochemical variables (Table 3), mean PTH was 128.6 pg/mL, albumin-corrected calcium was 10.6 mg/dL, ionized calcium was 1.37 mMol/L, and mean serum phosphate was in the low-normal range at 2.8 mg/dL. Mean 25(OH)D was optimal (31.1 ng/mL) and 111 patients (88.8%) were supplemented with vitamin D (104 with cholecalciferol - mean dosage 1554IU/day, 7 with calcifediol-mean dosage 0.266 mg/monthly) at the time of biochemical evaluation, furthermore renal function was preserved (mean eGFR 82.8 mL/min). Bone turnover markers were moderately increased (BAP 25.9 microg/L, CTX 0.710 ng/mL) and mean urinary calcium was 293 mg/24 h while urinary phosphate was 0.71 g/24 h. No significant differences in biochemical variables between degraded and non-degraded TBS patients were noted, except for urinary calcium (239 mg/24 h vs. 320 mg/24 h, *p* <.05) and eGRF (77 mL/min vs. 86 mL/min, *p* <.05), respectively. However, after analyzing these data using a regression model including age and BMI as covariates, the differences in urinary calcium excretion (*p* =.051) and eGFR (*p* =.193) were no longer significant between the two subgroups.

**Table 3:** Biochemical and hormonal characteristics of the study population according to TBS groups

Variables	Study population *N* = 125	TBS > 1.2 *N* = 84	TBS ≤ 1.2 *N* = 41
PTH [pg/mL]	128.6 ± 70.8 [37.0; 539.0]	120.9 ± 61.0 [37.0; 382.0]	144.5 ± 86.2 [41.0; 539.0]
Serum Albumin [g/l]	42.6 ± 2.5 [35.2; 46.9]	42.7 ± 2.6 [35.2; 46.9]	42.4 ± 2.1 [38.9; 46.7]
Serum Calcium [mg/dL]	10.9 ± 0.6 [9.3;13.0]	10.8 ± 0.6 [9.3; 12.8]	11.0 ± 0.6 [10.1; 13.0]
Serum Phosphate [mg/dL]	2.8 ± 0.5 [1.5; 4.2]	2.8 ± 0.5 [2.0; 4.1]	2.8 ± 0.5 [1.5; 4.2]
Serum Magnesium [mg/dl]	2.1 ± 0.2 [1.5; 3.1]	2.0 ± 0.2 [1.5; 2.4]	2.1 ± 0.2 [1.7; 3.1]
Urinary Calcium [mg/24 h]	293 ± 165 [18; 880]	320 ± 172^*^ [18; 880]	239 ± 138 [35; 580]
Urinary Phosphate [mg/24 h]	0.72 ± 0.31 [0.20; 2.10]	0.74 ± 0.29 [0.20; 1.50]	0.67 ± 0.35 [0.20; 2.10]
25(OH)D [ng/mL]	31.1 ± 12.5 [4.0; 86.0]	30.8 ± 12.1 [4.0; 73.0]	31.8 ± 13.3 [15.0; 86.0]
Vitamin D supplementation, n (%)	111 (88.8%)	77 (91.7%)	34 (82.9%)
CTX [ng/mL]	0.710 ± 0.572 [0.033; 3.210]	0.676 ± 0.522 [0.033; 2.303]	0.779 ± 0.663 [0.093; 3.210]
Bone Alkaline Phosphatase [microg/L]	25.9 ± 15.6 [6.7; 101.0]	24.9 ± 16.3 [6.7; 101.0]	28.0 ± 14.1 [10.4; 71.0]
Ionized Calcium [mMol/L]	1.368 ± 0.075 [1.100; 1.580]	1.36 ± 0.07 [1.26; 1.58]	1.38 ± 0.09 [1.10; 1.53]
Serum Creatinine [mg/dl]	0.81 ± 0.22 [0.50; 2.34]	0.8 ± 0.2 [0.5; 1.5]	0.8 ± 0.3 [0.5; 2.3]
eGFR [mL/min]	83 ± 17 [22; 112]	86 ± 16^*^ [36; 112]	77 ± 17 [22; 102]
Serum Calcium Min [mg/dl]	10.3 ± 0.5 [8.9;11.9]	10.2 ± 0.5 [8.9; 11.4]	10.4 ± 0.5 [9.4; 11.9]
Serum Calcium Max [mg/dl]	11.2 ± 0.7 [10.0; 16.5]	11.2 ± 0.8 [10.0; 16.5]	11.2 ± 0.6 [10.4; 13.4]
Albumin-corrected Serum Calcium [mg/dL]	10.6 ± 0.6 [9.4; 13.1]	10.6 ± 0.6 [9.4; 12.6]	10.8 ± 0.7 [9.7; 13.1]

BMI Body mass index; TBS, Trabecular bone score; 25(OH)D, 25-Hydroxyitamin D; CTX, C-terminal telopeptide of collagen type 1; eGFR, Estimated Glomerular Filtration Rate.

**P <.001 vs. Degraded TBS; *P <.05 vs. Degraded TBS.

§§P adjusted for age and BMI < 0.001 vs. Degraded TBS; § P adjusted for age and BMI < 0.05 vs. Degraded TBS.

## Discussion

TBS is an index derived from the grey scale variation in a two-dimensional projection obtained by DXA of the spine and allows an estimation of fracture risk based on the indirect assessment of bone microarchitecture [12]. The clinical contexts in which TBS can be useful are manifold and for this reason, several studies have been carried out concerning its use. Since 2012, TBS has been studied in the management of patients with primary hyperparathyroidism, however, more than 10 years have passed and the clinical utility of TBS in the decision-making process for parathyroid surgery still remains uncertain.

For the aforementioned reasons, our study aimed to better characterize bone quality in PHPT patients, as also stated in the research agenda of the latest international guidelines [16], and to clarify the usefulness of TBS in clinical practice, proposing it as a means for identifying a proportion of PHPT patients at high fracture risk who could benefit from parathyroidectomy and may not be captured by other clinical tools.

Analyzing the characteristics of the study population, it emerged that patients with degraded TBS had lower T-score values at all sites than patients with TBS > 1.2, thus underlining the concordance of TBS and DXA in identifying patients with a high fracture risk. Furthermore, in our study 68.8% of patients presented degraded or partially degraded TBS, while only 36.8% showed osteoporosis at any of the 3 sites. This evidence is in line with literature, as in the study by Muñoz-Torres et al. [17], in which 72 patients with PHPT were enrolled, 51.4% presented a degraded TBS (in this study degraded TBS was considered as ≤ 1.23) and 73.6% had a TBS < 1.31. Of these 72 patients, only 37.5% were osteoporotic according to the BMD values of the three sites. The study by Grigorie et al. [18] also agrees with our study, as out of 153 patients with PHPT 32% presented degraded TBS, 51% partially degraded and 17% preserved. This evidence suggests that TBS is able to detect a greater proportion of patients with an increased risk of fracture than BMD alone, precisely because of its ability to identify the qualitative degradation of bone microarchitecture. It should also be noted that 17 patients in our study had been taking oral bisphosphonates for less than two years (mean duration of therapy was 387 days). This did not impact BMD classification of the patients compared to the starting point of these medications (i.e. the diagnosis of osteopenia or osteoporosis remained stable).

Classifying patients according to lumbar spine BMD categories, osteoporotic patients showed lower TBS values compared to both osteopenic patients and to those with normal BMD. This finding is in partial agreement with the study by Munoz-Torres et al. [17], in which TBS values were lower in osteoporotic patients compared to normal BMD (1.16 ± 0.12 vs. 1.26 ± 0.17; *p* =.043), but not to osteopenia.

Analyzing the cohort of patients with osteoporosis at the third distal radius, a strong association was seen with low TBS (*p* =.003). The group of patients with TBS ≤ 1.2 had an almost two-fold greater incidence of osteoporosis at the distal third of the radius than patients with TBS > 1.2. This association supports a pathophysiological role of PTH excess both on cortical bone and trabecular bone. In any case, forearm DXA still remains essential in the management of PHPT patients because, as demonstrated by Castellano et al. [19], this assessment also increases the rate of patients with asymptomatic primary hyperparathyroidism meeting surgical criteria.

Another debated issue is the association between TBS and biochemical indices in patients with PHPT. There is conflicting data in the literature, with two studies [20, 21] showing that there were no associations between TBS and various biochemical indices. Two other studies contrasted this evidence, the first [22] showing an association between TBS and BAP values, and the second [23] showing an association between TBS and 25(OH)D values. In our study, there were no significant differences in biochemical indices between patients with degraded and not degraded TBS. In particular, after adjusting for age and BMI, no differences were noted in eGFR and urinary calcium values because as expected degraded TBS is more frequent in older patients who often show declined kidney function and consequently reduction of urinary calcium excretion due to aging and/or or concomitant known disease involving kidney function (e.g. obesity and arterial hypertension).

Considering the distribution of patients with degraded TBS in the various surgical indications, the most relevant evidence is the higher incidence of fragility fractures or morphometrical vertebral fractures in patients with degraded TBS compared to those with TBS > 1.2. In literature, many studies confirm this finding, such as the one conducted by Romagnoli et al. [24] or the one by Eller-Vainicher et al. [25]. TBS’s ability to identify patients at increased risk of vertebral fractures is well established and it has been demonstrated in patients with PHPT [26, 27] as well as in patients with post-menopausal osteoporosis [28, 29].

Within osteopenic patients the percentage of those with degraded TBS was significantly lower than that in osteoporotic patients. Reasoning on the basis of the current Italian [30] and international [16] guidelines, however, it is very important not to underestimate this proportion of osteopenic patients with degraded TBS, because they still have significant fracture risk but may not meet surgical criteria, unlike their counterparts with osteoporosis. This evidence is also supported by the Manitoba study [31] carried out in Canada in 2011, which stated that the risk of fracture in post-menopausal women with osteopenic BMD and degraded TBS is equal to or greater than that of women with osteoporosis.

We found that within the cohort of patients who did not meet the current surgical recommendations (*N* = 13), (*N* = 3) 23.1% presented a degraded TBS, and these patients had osteopenia. Thanks to this last piece of evidence, we understand the added value of performing TBS in clinical practice; in fact, by implementing its use in the clinical routine, we could identify a substantial proportion of patients with degraded TBS and osteopenia who would not otherwise be referred to surgery, but who nevertheless carry an increased fracture risk.

The strengths of this study were the single-center design and the exclusion of most drugs and disorders interfering with calcium-phosphorus metabolism or with bone quality. Also, all patients had a DXA performed with TBS at the 3 recommended sites (lumbar, hip, radius) with the same DXA machine, and underwent complete serum and urinary assessment in the same laboratory. The limitation of this study is the retrospective design as residual confounding might be present, as well as the cross-sectional analysis, as no follow-up information was provided to inform on the predictive power of TBS in the risk of incident fractures in PHPT. We also acknowledge a relatively small number of patients to draw definitive conclusions on the precise proportion of patients with degraded TBS alone and with no other surgical criteria. This last figure might be dependent on the study population examined.

## Conclusions

Our study found that TBS could be a useful tool in clinical practice for assessing the risk of fragility fractures in PHPT patients because of its ability to identify the qualitative bone degradation that DXA alone could not detect. In our population we confirmed the absence of correlations between TBS and biochemical indices, furthermore, degraded TBS well correlated with low cortical BMD at the forearm, reiterating the detrimental effect of PHPT on both cortical and trabecular bone. Finally, by implementing routine TBS measurement in addition to screening of bone and kidney complications, an estimated one-fifth of patients who would not otherwise meet the V International Workshop surgical recommendations presented degraded TBS (< 1.2). For this reason, TBS could be considered as a possible new indication for surgical treatment. In fact, this subgroup of patients could benefit from curative parathyroid surgery to reduce fracture risk regardless of other PHPT complications. However, further longitudinal studies with larger cohorts will be needed to confirm this hypothesis, to eventually include the combination of osteopenia and degraded TBS among the listed criteria to recommend parathyroid surgery.

## Data Availability

The datasets are available upon reasonable request to the Corresponding Author.
